# Update on preclinical models of cancer therapy‐related cardiac dysfunction: Challenges and perspectives. A scientific statement of the Heart Failure Association (HFA) of the ESC, the ESC Council of Cardio‐Oncology, and the ESC Working Group on Cellular Biology of the Heart

**DOI:** 10.1002/ejhf.3636

**Published:** 2025-03-11

**Authors:** Alessandra Ghigo, Pietro Ameri, Aarti Asnani, Edoardo Bertero, Rudolf A. de Boer, Dimitrios Farmakis, Arantxa González, Stephane Heymans, Borja Ibáñez, Teresa López‐Fernández, Alexander R. Lyon, Piero Pollesello, Amina Rakisheva, Konstantinos Stellos, Katrin Streckfuss‐Bömeke, Carlo Gabriele Tocchetti, Thomas Thum, Peter van der Meer, Eva Van Rooij, Piotr Ponikowski, Marco Metra, Giuseppe Rosano, Sophie Van Linthout

**Affiliations:** ^1^ Molecular Biotechnology Center Guido Tarone, Department of Molecular Biotechnology and Health Sciences University of Torino Turin Italy; ^2^ Cardiac, Vascular, and Thoracic Department IRCCS Ospedale Policlinico San Martino Genoa Italy; ^3^ Department of Internal Medicine University of Genova Genoa Italy; ^4^ Division of Cardiovascular Medicine Beth Israel Deaconess Medical Center and Harvard Medical School Boston MA USA; ^5^ Cardiovascular Institute, Thorax Center, Department of Cardiology Erasmus Medical Center Rotterdam The Netherlands; ^6^ Department of Cardiology Athens University Hospital Attikon, National and Kapodistrian University of Athens Medical School Athens Greece; ^7^ Program of Cardiovascular Diseases, Foundation for Applied Medical Research (CIMA), Department of Cardiology Clínica Universidad de Navarra, Instituto de Investigación Sanitaria de Navarra (IdiSNA), Universidad de Navarra Pamplona Spain; ^8^ Centro de Investigación Biomédica en Red Cardiovascular (CIBERCV), Instituto de Salud Carlos III Madrid Spain; ^9^ Department of Cardiology CARIM Cardiovascular Research Institute Maastricht, Maastricht University Medical Centre Maastricht The Netherlands; ^10^ Centre of Cardiovascular Research, University of Leuven Leuven Belgium; ^11^ Centro Nacional de Investigaciones Cardiovasculares Madrid Spain; ^12^ Cardiology Department IIS‐Fundación Jiménez Díaz Hospital Madrid Spain; ^13^ Cardiology Department La Paz University Hospital Madrid Spain; ^14^ IdiPAZ research Institute Madrid Spain; ^15^ Cardiology Department Quironsalud Madrid University Hospital Madrid Spain; ^16^ Cardio‐Oncology Centre of Excellence, Royal Brompton Hospital London UK; ^17^ Content and Communication, Branded Products, Orion Pharma Espoo Finland; ^18^ Department of Cardiology City Cardiology Center Almaty Kazakhstan; ^19^ Department of Cardiology Qonaev City Hospital Almaty Kazakhstan; ^20^ Department of Cardiovascular Research Medical Faculty Mannheim, Heidelberg University Mannheim Germany; ^21^ German Centre for Cardiovascular Research (DZHK), Partner Site Heidelberg/Mannheim Mannheim Germany; ^22^ Biosciences Institute, Vascular Biology and Medicine Theme, Faculty of Medical Sciences, Newcastle University Newcastle upon Tyne UK; ^23^ Medizinische Klinik I, Department of Cardiology, Angiology, Haemostaseology and Intensive Care Medicine University Hospital Mannheim of the Heidelberg University Mannheim Germany; ^24^ Clinic for Cardiology and Pneumology, Georg‐August University Göttingen Göttingen Germany; ^25^ Medical Clinic I, Cardiology and Angiology, Justus‐Liebig‐University Giessen Germany; ^26^ Institute of Pharmacology and Toxicology, University of Würzburg Würzburg Germany; ^27^ Comprehensive Heart Failure Center Würzburg Würzburg Germany; ^28^ Department of Translational Medical Sciences (DISMET) Center for Basic and Clinical Immunology Research (CISI), Interdepartmental Center of Clinical and Translational Sciences (CIRCET), Interdepartmental Hypertension Research Center (CIRIAPA), Federico II University Naples Italy; ^29^ Institute of Molecular and Translational Therapeutic Strategies (IMTTS), Hannover Medical School Hannover Germany; ^30^ Department of Cardiology University Medical Center Groningen, University of Groningen Groningen The Netherlands; ^31^ Hubrecht Institute, Royal Netherlands Academy of Arts and Sciences (KNAW) and University Medical Center Utrecht Utrecht The Netherlands; ^32^ Department of Cardiology University Medical Center Utrecht Utrecht The Netherlands; ^33^ Center for Heart Diseases, University Hospital, Wroclaw Medical University Wroclaw Poland; ^34^ Institute of Cardiology, ASST Spedali Civili, Department of Medical and Surgical Specialties Radiological Sciences, and Public Health, University of Brescia Brescia Italy; ^35^ Department of Human Sciences and Promotion of Quality of Life San Raffaele Open University of Rome Rome Italy; ^36^ Cardiology, San Raffaele Cassino Hospital Cassino Italy; ^37^ Berlin Institute of Health at Charité‐Universitätsmedizin Berlin, BIH Center for Regenerative Therapies (BCRT) Berlin Germany; ^38^ German Center for Cardiovascular Research (DZHK), Partner site Berlin Berlin Germany

**Keywords:** Cardiotoxicity, Cancer, Anticancer therapy, Immunotherapy, Preclinical models

## Abstract

New anticancer therapies with potential cardiovascular side effects are continuously being introduced into clinical practice, with new and often unexpected toxicities becoming apparent only after clinical introduction. These unknown toxicities should be identified and understood beforehand to better prepare patients and physicians, enabling the implementation of effective treatments. Therefore, there is a crucial need for appropriate preclinical models to understand the biological basis of their cardiotoxicity. This scientific statement summarizes the preclinical models hitherto used, from *in vitro* two‐ and three‐dimensional human systems to small and large animals, to pinpoint the molecular mechanisms behind the cardiotoxicity of new‐generation anticancer therapies, particularly immunotherapies, and to develop potential cardioprotective strategies. Furthermore, it discusses how preclinical models have contributed to the provocative concept of heart failure being potentially tumorigenic and how the discovery of drugs with both anticancer and cardioprotective actions has revealed a common mechanistic basis for heart failure and cancer. Finally, it discusses the existing gaps between preclinical models and clinical observations in patients, how these discrepancies affect regulatory pathways and the drug development process in cardio‐oncology and provides recommendations for closing these gaps.

## Introduction and scope

**Figure 1:**
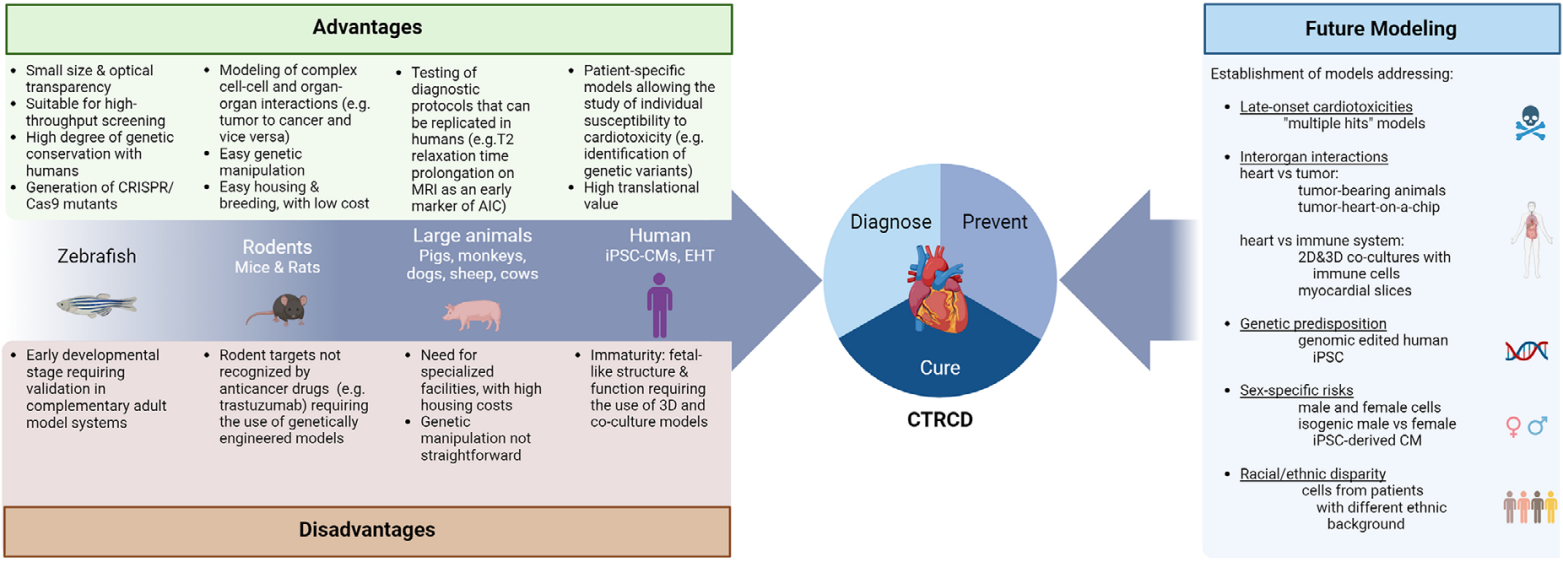
Current preclinical models of cancer therapy‐related cardiac dysfunction (CTRCD) and recommended future model systems addressing gaps between current preclinical models and clinical observations. Left panel: Overview of preclinical model systems across different species utilized in studies aimed at identifying new approaches for diagnosing, preventing, or curing CTRCD. The pros and cons of each model are highlighted. Right panel: Recommended future model systems addressing current gaps in preclinical modelling of CTRCD. 2D, two‐dimensional; 3D, three‐dimensional; AIC, anthracycline‐induced cardiotoxicity; CM, cardiomyocyte; EHT, engineered heart tissue; iPSC, induced pluripotent stem cell; MRI, magnetic resonance imaging. (The figure has been created with Biorender.com).

Cancer therapy‐related cardiovascular toxicity is an important clinical problem. Medications with established cardiotoxicity, but also high efficacy, like anthracycline chemotherapy and human epidermal growth factor receptor 2 (HER2)‐targeted therapies, are widely used in clinical practice. In addition, many new anticancer therapies with potential cardiac side effects are continuously introduced into clinical practice. Cancer therapy‐related cardiovascular toxicity can manifest as a wide range of potentially life‐threatening complications, including cancer therapy‐related cardiac dysfunction (CTRCD), arrhythmias, and myocarditis.^1^ Preclinical models, including two‐ (2D) and three‐dimensional (3D) cellular systems as well as small and large animals, have proven to be indispensable tools for elucidating the mechanistic pathways responsible for the cardiac side effects of traditional anticancer therapies.^2^ This scientific statement summarizes studies from the past 5 years (2019–2024) that have utilized these models to advance the mechanistic understanding of the cardiotoxicity of new generation oncological treatments, and of the complex interaction between cancer and heart failure (HF). It also discusses the current gaps between preclinical modelling and clinical observations in patients (*Figure* *1*), how these gaps affect regulatory pathways and the overall feasibility of the drug development process, and provides recommendations for closing these gaps. While anticancer therapies can have various adverse effects on the cardiovascular system, such as arrhythmias, hypertension, and thrombosis, and others, this review will specifically concentrate on the mechanisms leading to cardiac dysfunction and HF, aiming to provide a detailed analysis while maintaining a clear and targeted scope.

## Mechanisms and clinical manifestation of cardiac dysfunction of conventional and emerging cancer therapies

### Conventional therapies

Conventional cancer treatments with known cardiotoxic effects include anthracyclines, anti‐HER2 therapies, tyrosine kinase inhibitors and radiotherapy. Anthracycline‐induced cardiotoxicity (AIC), which irreversibly progresses from asymptomatic left ventricular dysfunction to HF,^1^, ^3^, ^4^ is the most extensively studied form of cardiotoxicity due to the availability of established preclinical models across various species. Studies using zebrafish, rodents, pigs and human cells have identified increased reactive oxygen species, activation of the DNA damage response, impaired autophagy, and mitochondrial dysfunction as key drivers of toxicity in cardiomyocytes^5^ and other cardiac cell types.^6^, ^7^

Another example is trastuzumab, a humanized monoclonal antibody targeting the ERBB2/HER2 receptor tyrosine kinase. Unlike anthracycline regimens, trastuzumab‐induced cardiotoxicity is highly reversible, but can amplify and exacerbate AIC when co‐administered.^1^ Initial insights into the cardiotoxicity of anti‐HER2 therapies came from conditional ErbB2 mouse mutants that develop severe dilated cardiomyopathy^8^ and from mice with cardiac‐specific ErbB2 overexpression that are protected against doxorubicin‐induced oxidative stress.^9^ Human‐induced pluripotent stem cells (iPSCs) subsequently extended our mechanistic understanding of trastuzumab cardiotoxicity beyond inhibition of the pro‐survival NRG1/ErbB pathway in cardiomyocytes^10^ and revealed alterations in metabolic pathways^11^ and in the interaction between cardiomyocytes and vascular endothelial cells.^12^

Tyrosine kinase inhibitors (TKIs) also cause cardiac side effects.^1^ Although some are specific, the majority of clinically licensed TKIs inhibit multiple kinases. Targeting BCR‐ABL with ponatinib causes cardiomyopathy, HF, and vascular occlusion,^13^ with mitochondrial toxicity,^14^ integrated stress response activation^14^ and S100A8/A9‐NLRP3‐interleukin (IL)‐1β‐mediated inflammation^15^ identified as key mechanisms in human iPSC and murine models.^14^, ^15^ Sunitinib, a multi‐targeted TKI in the class of vascular endothelial growth factor (VEGF) TKIs that causes hypertension and left ventricular dysfunction,^1^ similarly induces mitochondrial dysfunction in engineered cardiac microtissues.^16^ Bruton's tyrosine kinase inhibitors (BTKi) have revolutionized the treatment of B‐cell malignancies including chronic lymphocytic leukaemia and mantle cell lymphoma. Patients treated with BTKi have an increased risk of arterial hypertension, atrial fibrillation and CTRCD. Insights from studies in mice lacking an active Bruton's tyrosine kinase suggest that part of the arrhythmia risk associated with the BTKi ibrutinib is related to an off‐target blockade of C‐terminal Src kinase.^17^ However, more specific second‐generation BTKi also have an increased risk of atrial fibrillation and hypertension,^18^ suggesting that direct Bruton's tyrosine kinase inhibition may be relevant in the pathogenesis of atrial fibrillation and hypertension in humans.

Radiation‐induced heart disease, which typically manifests several years to decades after therapy, includes coronary artery disease, valvular heart disease, pericardial disease, conduction abnormalities, and HF.^1^, ^19^ Studies in myocytes and mice exposed to radiation that mimics the dose, fractionation, and beam delivery of clinical radiotherapy protocols have identified cardiac fibrosis^20^ and cardiomyocyte Ca^2+^ mishandling^21^ as key pathogenic events in radiation‐induced heart disease.

Two decades of preclinical research have revealed the molecular underpinnings of the cardiotoxicity of most conventional oncological treatments, as extensively reviewed in Morelli *et al*.^22^ However, the potential interactions between cancer therapies in multimodal regimens, as well as the effects of cardiotoxic treatments against the backdrop of pre‐existing cardiovascular risk factors or disease, have been insufficiently addressed in preclinical modelling and should be the focus of future studies.

### Immune checkpoint inhibitors

The therapeutic landscape in cancer has been profoundly transformed by the advent of immune checkpoint inhibitors (ICIs), a class of monoclonal antibodies that boost the anticancer immune response by targeting immune‐cell surface receptors.^23^ ICIs target various co‐stimulatory signalling molecules on T lymphocytes and antigen‐presenting cells, such as cytotoxic T‐lymphocyte antigen 4 (CTLA‐4), programmed cell death protein 1 (PD‐1), and its ligand (PD‐L1), thereby breaking the immune tolerance of T cells against cancer cells.^24^

Despite their proven efficacy,^25^, ^26^ the use of ICIs has led to the occurrence of several immune‐related adverse events (irAEs) caused by the hyperactivation of the immune system against non‐cancerous tissues.^27^ Although irAEs depend on the type of cancer, the ICI used, the duration of exposure and the dose administered, ICI immunotherapy is associated with an increased risk of cardiac events, including myocarditis, arrhythmias, HF, and cardiovascular death.^28^, ^29^, ^30^, ^31^

Animal models have been instrumental in deciphering the exact pathophysiological mechanisms underlying the cardiovascular toxicities of ICIs (extensively reviewed in Tocchetti *et al*.^31^ and Gergely *et al*.^32^). Studies in mice with genetic deletion of immune checkpoints like *Pdcd1* and *Ctla4*, which are the murine equivalents of the genes encoding human PD‐1 and CTLA‐4, have shown that ICI cardiovascular toxicity is associated with increased inflammation. This is secondary to infiltration of macrophages^33^ and cytotoxic CD8^+^ T cells specific for α‐myosin^34^ into the heart. In mice with monoallelic loss of *Ctla4* combined with a complete genetic absence of *Pdcd1*, myocardial infiltration was associated with severe electrocardiographic abnormalities and left ventricular dysfunction observed in some ICI‐treated patients.^35^ Interestingly, in this murine model, which closely recapitulates the clinical and pathological features of ICI‐associated myocarditis, intervention with CTLA4‐Ig (abatacept) ameliorated disease progression.^35^ This was supported by a case series of patients receiving abatacept, which successfully attenuated the fulminant course of ICI myocarditis, although the optimal dosing for this indication and pharmacokinetics in humans are complex and have not been definitively established.^35^

Preclinical models will be essential to validate new strategies, like fasting mimicking diet cycles,^36^ to protect the heart from the adverse effects of immunotherapy. At the same time, these models should be used to address current knowledge gaps. The observation that only 1–1.5% of all patients receiving ICI develop severe cardiovascular toxicity,^27^, ^31^ along with the variable progression of ICI‐mediated myocarditis among individuals, suggests that additional genetic and environmental factors must contribute to the manifestation of this condition. Indeed, traditional cardiovascular risk factors such as diabetes, hypertension, and dyslipidaemia do not predict which patients are at risk. In contrast, patients undergoing combination ICI therapy, those with pre‐existing autoimmune diseases, and those with specific cancers, such as thymic cancers, are at increased risk.^31^, ^37^ This indicates that the immune phenotype plays a critical role, presenting a challenge for translational models due to significant species‐related differences in immunobiology. Therefore, preclinical models which recapitulate the human immune response are critical to decipher the patient‐specific response towards ICI.

### CAR‐T and TCR‐T cells

Chimeric antigen receptor T cell (CAR‐T) therapy is a form of immunotherapy in which patient's own T cells are genetically engineered to recognize and eliminate cancer cells. CAR‐T cells are generated by inducing the expression of an artificial protein, the chimeric antigen receptor, consisting of a T cell signalling domain and an antigen recognition domain that selectively targets a cancer‐specific antigen. CAR‐T therapy has drastically improved the prognosis of previously incurable haematologic malignancies, particularly those derived from B cells.^38^ The potential of CAR‐T cells to treat a range of conditions beyond cancer, including autoimmune diseases, chronic infections, cardiac fibrosis, senescence‐associated diseases and other conditions, is also being explored in preclinical models.^39^

The therapeutic efficacy of CAR‐T therapy is compromised by serious clinical complications, the most common being cytokine release syndrome (CRS), a systemic inflammatory syndrome characterized by immune cell activation and high levels of circulating cytokines.^40^ CRS develops on average 5–7 days after CAR‐T infusion and its manifestation varies in severity from fever, with or without constitutional symptoms, to a potentially life‐threatening condition.^41^ Evidence from preclinical mouse models^42^ has demonstrated the importance of IL‐1β^43^ and IL‐6^44^ in the pathophysiology of CRS, and the benefit of IL‐1 receptor and IL‐6 blockade in ameliorating CRS. To date, several clinical trials are underway testing the efficacy of CAR‐Ts combined with IL‐1 receptor antagonism,^45^ while IL‐6 receptor blockade with tocilizumab has shown benefit in patients with moderate or severe CRS.^46^

Cardiovascular toxicity is a frequently reported complication of CAR‐T therapy. CAR‐T‐related cardiovascular toxicity often occurs in the context of CRS,^47^, ^48^ and the risk of cardiovascular events correlates with CRS severity.^49^ The most common manifestations are new‐onset HF and supraventricular tachyarrhythmias, but CAR‐T‐related severe CRS and cardiac dysfunction can also lead to refractory cardiogenic shock and death (*Table* *1*).^47^, ^49^, ^50^, ^51^, ^52^, ^53^, ^54^, ^55^, ^56^ However, it is important to emphasize that these clinical data are derived from retrospective analyses, which are inherently susceptible to bias and may not fully reflect real‐world clinical scenarios. The pathophysiology of CAR‐T‐related cardiovascular toxicity has not been investigated so far in animal models of CRS,^57^ but myocardial depressing effects of cytokines like IL‐6 have been described in mice.^58^

**Table 1:** Frequency of cytokine release syndrome and cardiovascular events in adult patients receiving CAR‐T therapy

Study	No. of patients and type of malignancy	CRS	LVSD/AHF	Arrhythmias	CV death
Alvi *et al*.^47^	*n* = 137 DLBCL 83 (60.6), follicular lymphoma 36 (26.3), MM 11 (8.0), other 7 (5.1)	81 (59%)	6 (4.4) AHF, of which 4 new‐onset	5 (3.6), of which 3 AF and 2 SVT	6 (4.4)
Lefebvre *et al*.^49^	*n* = 145 ALL 36 (24.8), CLL 66 (45.5), DLBCL 43 (29.7)	176 events in 104 pts (71.7)	22 AHF events in 21 pts (14.5)	12 episodes of AF in 11 pts (7.6), 1 SVT, 1 NSVT	2 (13.8)
Ganatra *et al*.^50^	*n* = 187 DLBCL 137 (73.3), follicular lymphoma 35 (18.7), other B‐cell malignancies 15 (8.0)	155 (82.9)	12 (10.3% of 116 pts with echo) LVSD, of which 6 AHF events	10 (5.3), unspecified	3 (1.6)
Goldman *et al*.^51^	*n* = 2657 NHL 1777 (83.7), ALL 306 (14.4), other 39 (1.8)	1457 (54.8)	Cardiomyopathy 69 (2.6)	74 (2.8), of which 55 AF, 10 VT, 4 VF	NA
Qi *et al*.^52^	*n* = 126 MM 78, NHL 25, ALL 23	103 (81.7)	15 (11.9) AHF, 1 (0.8) LVSD	7 (5.6), unspecified	2 (1.6)
Lee *et al*.^53^	*n* = 78 RRMM 78 (100)	16 (20.5) grade 2 or above	4 (5.1) LVSD, of which 1 (1.3) AHF	11 (14.1), of which 8 AF/flutter and 3 NSVT	1 (1.3)
Lefebvre *et al*.^54^	*n* = 44 Lymphoma 43 (97.7), ALL 1 (2.3)	24 events in 23 (52.3) pts	1 (2.3) AHF with preserved LVEF	1 (2.3) AF	None
Mahmood *et al*.^55^	*n* = 202 B‐cell lymphoma 143 (70.8), ALL 59 (29.2)	139 (68.8)	26 (12.8) AHF, of which 2 (1.0) cardiogenic shock	37 (18.3) atrial arrhythmias	1 (0.5)
Korell *et al*.^56^	*n* = 137; B‐cell lymphoma 104 (75.9), B‐ALL 19 (13.8), MM 14 (10.2)	75 (60.1)	4 (2.9) LVSD	5 (3.6) AF	None

AF, atrial fibrillation; AHF, acute heart failure; ALL, acute lymphoblastic leukaemia; CRS, cytokine release syndrome; CV, cardiovascular; DLBCL, diffuse large B‐cell lymphoma; LVEF, left ventricular ejection fraction; LVSD, left ventricular systolic dysfunction; MM, multiple myeloma; NA, not available; NHL, non‐Hodgkin's lymphoma; NSVT, non‐sustained ventricular tachycardia; pts, patients; RRMM, relapsed and refractory multiple myeloma; SVT, supraventricular tachycardia; VF, ventricular fibrillation; VT ventricular tachycardia.

Cardiac safety concerns have also arisen with T cell receptor (TCR) T cell (TCR‐T) therapies, immunotherapies which use engineered TCR‐Ts expressing cancer antigen‐specific TCRs to redirect T cells against tumour cells.^59^ Unlike CAR‐T, which target cell surface antigens, TCR‐T recognize epitopes presented by the major histocompatibility complex (MHC). Although TCR‐T is less likely to induce CRS compared with CAR‐T therapy,^59^ two cases of fatal myocarditis due to TCR‐T targeting MHC class I‐restricted epitopes derived from the melanoma‐associated antigen‐3 (MAGE‐A3) have been reported.^60^, ^61^ Mechanistically, these effects were linked to cross‐reactivity against titin, as demonstrated in iPSC‐derived cardiomyocytes (iPSC‐CMs) exposed to MAGE‐A3‐transduced T cells.^60^, ^61^

In conclusion, CAR‐T and TCR‐T therapies are associated with cardiac events stemming from cardiotoxic effects of interleukins in the context of CRS, and off‐target cytotoxic effects of engineered T cells, respectively. A deeper understanding of these pathomechanisms requires further investigation in appropriate preclinical models.^57^

## Preclinical models to study cancer therapy‐related cardiac dysfunction

### *In vitro* models

#### Induced pluripotent stem cell‐derived cardiomyocyte and non‐cardiomyocyte cells in 2D and 3D tissue models

For a long time, there was a lack of human cardiomyocyte culture models that could be used to understand the molecular and cellular physiology of CTRCD; as a result, preclinical cardio‐oncology research relied primarily on rodent cardiomyocytes as an *in vitro* model. More recently, the development of 2D and 3D culture systems has enabled the use of iPSC‐CMs as a screening platform for cardiotoxic effects of cancer therapies, to predict cardiotoxicity risk,^62^, ^63^ or to analyse the underlying pathophysiology.^10^, ^11^ By generating human iPSC‐CM and non‐cardiomyocyte cell types, it is possible to establish patient‐specific disease models to define cardiotoxicities at the individual and population levels. In addition, patient‐specific iPSC models can be used to identify and validate single nucleotide polymorphisms associated with CTRCD, ultimately guiding the design of clinical trials.^64^

Healthy control iPSC‐CMs have initially been used to demonstrate general cardiotoxic effects of anthracyclines, such as apoptosis, reactive oxygen species production, mitochondrial dysfunction, and electrophysiological and Ca^2+^ changes,^65^, ^66^ and led to the identification of telomerase overexpression as a therapeutic option in CTRCD.^67^ On the other hand, patient‐specific iPSC models have been shown to recapitulate the predisposition of individual breast cancer patients to low doxorubicin‐induced cardiotoxicity^62^ or trastuzumab‐induced cardiac dysfunction.^11^ Another patient‐specific stem cell model of CTRCD demonstrated that patients with aggressive B‐cell lymphoma are persistently more susceptible to detrimental effects of high doxorubicin treatment with hyperactivation of Ca^2+^/calmodulin‐dependent protein kinase II δ (CaMKIIδ), resulting in arrhythmogenic triggers and cellular dysfunction.^68^ Furthermore, iPSC‐CMs generated from paediatric oncology patients with acute anthracycline cardiotoxicity identified cardioprotective micro^69^ and circular^70^ RNAs. Finally, a stem cell model of paediatric CTRCD patients allowed the identification of genomic variants of drug transporters associated with doxorubicin‐induced cardiotoxicity, thereby confirming the evidence for a genetic predisposition to CTRCD,^71^ including several somatic and systemic mutations that trigger the development of CTRCD.^68^, ^72^

Although iPSC‐CMs hold great promise as an *in vitro* research tool for understanding the cardiotoxicity of oncological drugs, they also differ significantly from adult cardiomyocytes in that they are structurally and functionally immature, resembling foetal cardiomyocytes.^73^ This immaturity can be partially corrected by embedding the iPSC‐CMs in a 3D environment, such as the so‐called dynamic engineered heart tissue model^74^ or engineered heart myocardium.^75^ In addition to promoting maturation, as shown by a faster conduction velocity, better alignment, and increased contractile power, this also provided a method to analyse iPSC‐CM behaviour in a more physiological environment and the possibility to include other cell types, including endothelial cells and cardiac fibroblasts. Exposing dynamic engineered heart tissues or engineered heart myocardium to anthracyclines resulted in tissue dilatation and reduced contractile force, both key features of the clinical phenotype of AIC.^68^, ^76^ Furthermore, these 3D dynamic‐engineered cardiac tissues could recapitulate the induction of senescence observed in the hearts of patients with severe doxorubicin‐induced cardiotoxicity. However, pharmacological inhibition of senescence did not show any functional improvement in these models, calling into question the use of senolytic drugs as a potential cardioprotective therapy.^76^ Other maturation strategies for iPSC‐CMs currently being explored include long‐term culture, mechanical and/or electrical stimulation, 3D cardiac organoids of different cell types, the addition of anti‐inflammatory macrophages^77^ and combinatorial approaches.^78^, ^79^ While the majority of the studies with CTRCD stem cell models have focused specifically on autonomic iPSC‐CM processes, the interaction with other relevant (cardiovascular) cell types, including immune cells, and their integration into 3D engineered tissue systems, should also be considered. The inclusion of multicellular models into preclinical studies of CTRCD is expected to uncover previously unexplored aspects of the disease. Beyond immaturity and limited ability to reproduce multicellular communications, iPSC‐CMs face additional challenges, including subtype specification, scarce interlaboratory reproducibility, labour‐intensive protocols, and high costs.^80^ Moreover, the reprogramming, culture, and differentiation of human iPSCs demand significant time and resources, limiting the feasibility of large‐scale research for non‐established iPSC laboratories. While substantial progress has been made,^81^, ^82^ further efforts are needed to optimize cardiomyocyte differentiation protocols, prioritizing simplicity, reproducibility, and cost‐effectiveness, to enable broader application.

#### Cardiac slices

Although iPSC models have revolutionized our understanding of CTRCD, they still do not fully emulate the complex cellular composition of human heart tissue. Myocardial slices – thin, living sections of heart tissue – offer a unique advantage for studying cardiac function and drug effects in a context that closely resembles the native tissue environment. Using a biomimetic culture system, pig and human heart slices can remain completely viable and functionally and structurally intact for 6 days in culture, also enabling the assessment of contraction and relaxation kinetics on isolated single myofibrils after culture.^83^, ^84^ To date, canine, porcine, and human myocardial slices have been used to study the cardiotoxicity of doxorubicin, trastuzumab, and sunitinib. Heart slices effectively recapitulate the expected toxicity of doxorubicin^85^, ^86^ and trastuzumab,^86^ similar to human iPSC‐CMs, but are superior in detecting sunitinib cardiovascular toxicity at clinically relevant concentrations.^86^ Therefore, these systems should be considered for validating translational findings from other preclinical models in future studies.

### *In vivo* models

#### Zebrafish

The zebrafish is an *in vivo* whole organism model that has been used for decades to study the mechanisms of cardiovascular disease (CVD), as well as cancer growth and metastasis. In cardio‐oncology, zebrafish have been used to identify new small molecule therapies that protect against doxorubicin‐induced cardiomyopathy without compromising anti‐tumour activity.^87^ Zebrafish models have also been developed to study cardiovascular toxicities associated with cyclophosphamide^88^ and VEGF inhibitors.^89^, ^90^ Zebrafish have the following strengths when compared to *in vitro*, rodent, or large animal models: (1) small size and optical transparency in the embryonic stage, facilitating high throughput screening with direct cardiac imaging; (2) high degree of genetic conservation between zebrafish and humans, particularly for metabolic and cell death‐related pathways; (3) advances in genome editing technology, allowing rapid and efficient generation of CRISPR/Cas9 mutants; (4) studies at scale with large numbers and significantly lower cost compared to rodent models. One disadvantage is the early developmental stage required for most high‐throughput applications. As a result, validation of new molecular mechanisms discovered in zebrafish embryos typically requires the use of complementary adult model systems.

#### Rodents

Rodents are the most widely used preclinical model in cardio‐oncology research. CTRCD can be easily recapitulated by treating rats and mice with the anticancer drug according to protocols that closely mimic human therapeutic regimens in terms of route of administration, dosage and pharmacokinetic profile.^2^ Over the past decades, these models have been instrumental in identifying the mechanisms behind the cardiotoxicity of conventional chemotherapies, primarily anthracyclines, and in discovering potential cardioprotective strategies.^91^ However, direct treatment of rodents with anticancer therapies does not always effectively reproduce the associated cardiotoxicity, as seen with trastuzumab and other anti‐HER2 antibodies, because these drugs are unable to recognize the murine counterpart of the HER2 receptor.^92^ Homozygous human HER2 transgenic mice do not represent a valuable alternative as they are embryonic lethal.^93^ Consequently, most of the knowledge on the cardiotoxicity of anti‐HER2 therapies has been obtained from mice with cardiomyocyte‐specific ErbB2 deletion or overexpression.^8^, ^9^ These studies, along with later investigations involving the genetic deletion of immune checkpoints, exemplify the strengths of mouse genetics in advancing our understanding of the mechanistic basis of cardiotoxicity. Furthermore, this highlights the added value of murine models, particularly compared to large animals, where genetic manipulation is more challenging.

Another significant advantage of rodent models over human *in vitro* systems is the ability to assess the impact of novel cardioprotective candidates on the anti‐tumour activity of cancer therapies. For example, xenotransplantation of cells derived from cutaneous melanoma, a prototypical cancer type treated with immunotherapy, originally led to the identification of tumour necrosis factor (TNF)‐α blockade, but not CD8^+^ T cell depletion, as a means of preventing ICI‐mediated myocarditis without compromising the anticancer efficacy of the anti‐PD1 therapy.^94^ Nevertheless, despite this positive preclinical evidence, caution is advised against the use of TNF‐α antibodies for ICI myocarditis and HF,^1^ as clinical studies in HF patients have shown neutral or even negative outcomes.^95^ In addition to xenotransplantation of solid tumours into syngeneic mice, the use of patient‐derived xenografts (PDX) can further strengthen the translational value of the study. PDX generated using tissue from a woman with triple‐negative breast cancer, and implanted in severe combined immunodeficiency mice, demonstrated that a small molecule allosteric inhibitor of the pro‐apoptotic molecule BAX can provide cardioprotection, without compromising the efficacy of doxorubicin in reducing the burden of breast cancer *in vivo*.^96^ However, a major disadvantage of xenografts in immunodeficient mice is the inability to model interactions of the immune system with the tumour and the cardiovascular system. In this respect, genetic models with spontaneous tumour growth represent a valuable alternative. For example, using *ErbB2/Neu* transgenic mice, it has been shown that inhibition of PI3Kγ effectively protects against AIC, while inducing anti‐tumour immune responses, leading to reduced mammary cancer growth.^97^

In summary, although novel human *in vitro* models of CTRCD are emerging, rodents remain invaluable for studying complex cell–cell and organ–organ interactions, including tumour–heart interactions as well as the involvement of the immune system.

#### Large animals

Despite their scientific value, murine models differ significantly from human anatomy and physiology, limiting the straightforward translation to the clinic. Because the hearts of large mammals have increased similarity to humans, diagnostic and therapeutic strategies that prove beneficial in large animal studies are more likely to succeed in clinical trials than those tested only in small animals.^98^ Several large animal models of CTRCD (e.g. pigs, primates, sheep, cows, dogs) have been reported,^99^, ^100^, ^101^, ^102^, ^103^, ^104^, ^105^ most of which test for cardiac side effects of anthracyclines. Large animal models offer the opportunity to test diagnostic protocols that can be replicated in humans using exactly the same equipment. One example is the discovery of T2 relaxation time prolongation on magnetic resonance imaging as a very early marker of anthracycline cardiotoxicity in pigs and monkeys,^102^, ^104^ which was later validated in clinical studies.^106^, ^107^

Large animal models are also useful when testing potential cardioprotective therapies prior to clinical trials. Through the use of these models, there are examples of some therapies that have been withdrawn from the translational portfolio because they did not show any benefit in a large animal model.^100^ A successful example of this strategy is the testing of remote ischaemic conditioning as an intervention to prevent AIC. This non‐invasive intervention was tested in a porcine model of AIC and showed that its use prior to each doxorubicin injection resulted in a significant improvement in left ventricular ejection fraction through a mechanism involving mitochondrial protection.^108^ These results in the pig model have served as basis for the execution of the ongoing RESILIENCE trial (NCT05223413), which is investigating the benefits of weekly remote ischaemic conditioning in a population of cancer patients at high risk of AIC.

Despite their undeniable advantages, large animal models present significant challenges, including high costs, specialized infrastructure and handling requirements, and longer lifespans and gestation periods. Additionally, genetic manipulation in these animals is complex and may raise ethical concerns.^98^ Consequently, small animals, like murine models, remain the preferred choice in preclinical research, including in the field of cardio‐oncology.

## Mechanisms and preclinical models of reverse cardio‐oncology

Recent studies suggest that the relationship between CVD and cancer extends well beyond CTRCD. Community‐based cohort studies have shown that individuals with HF are at increased risk of cancer incidence and mortality compared with the general population, a phenomenon referred to as ‘reverse cardio‐oncology’.^109^, ^110^, ^111^ Murine models, particularly tumour xenografts, have been instrumental in demonstrating that the relationship between cancer and HF is not merely a clinical association, but rather a causal one. Landmark studies first demonstrated that HF, induced by permanent ligation of the left anterior descending coronary artery or by pressure overload‐induced left ventricular remodelling via transverse aortic constriction, leads to increased tumour burden in a mouse model of spontaneous intestinal adenomatosis,^112^, ^113^ independent of haemodynamic changes.^113^ Subsequent studies have confirmed these findings, showing that cardiac remodelling from left anterior descending coronary artery ligation, transverse aortic constriction, ATF3 overexpression, or prolonged low‐dose phenylephrine infusion promotes proliferation, and in some cases metastatic dissemination, of MMTV‐PyMT or 4T1 mammary cancer and Lewis lung carcinoma cells (*Table* *2*).^114^, ^115^, ^116^, ^117^, ^118^, ^119^, ^120^, ^121^

**Table 2:** Preclinical models in reverse cardio‐oncology studies

Cardiac intervention	Cancer model	Strain, sex	Effects of intervention		Main identified pathway	Effects on tumour cells (*in vitro*)	Ref.
			On the heart	On the tumour			
TAC (vs. sham surgery)	ApcMin/J mouse (intestinal adenoma)	Male	LVH, fibrosis	↑ tumour growth	Not provided (suggestion of fibrotic factors)	Not studied	^112^
TAC (vs. sham surgery)	MMTV‐PyMT (mammary cancer)	C57Bl/6, female	LVH, LVSD, no fibrosis	↑ tumour growth and proliferation	–	↑ proliferation of MMTV‐PyMT and LCC cells upon incubation with serum from TAC‐operated mice ↑ proliferation of MMTV‐PyMT and LCC cells upon incubation with POSTN	^114^
	i.v. MMTV‐PyMT cells (cancer metastases)			↑ lung metastases			
	LCC cells into the s.c. tissue of the flank (lung cancer)	C57Bl/6, male	LVH, LVSD, no fibrosis	↑ tumour growth and proliferation	Cardiac remodelling → tumorigenic factors (CTGF, POSTN)		
	i.v. LCC cells (cancer metastases)		–	↑ lung metastases			
	MMTV‐PyMT cells into the mammary fat (mammary cancer)	NOD/SCID, female	LVH, LVSD, no fibrosis	↑ tumour growth	–	–	
	MMTV‐PyMT cells into the mammary fat (mammary cancer)	JDP2xATF3 female	No cardiac remodelling	= tumour growth	= CTGF and periostin levels	–	
TAC (vs. sham surgery)	LCC cells into the sc tissue of the flank (lung cancer)	Postn^−/−^ C57Bl/6, male	LVH, LVSD	↑ tumour growth	Inflammatory and tumorigenic cytokines, FN = serum POSTN	↑ proliferation of MMTV‐PyMT and LCC cells upon incubation with serum from Postn^−/−^ mice	^115^
	Renca cells into the kidney (renal cancer)	BALB/c, NS	LVH, LVSD	= tumour growth		Low expression of ITGB1 in Renca cells	
	MMTV‐PyMT cells into the mammary fat (mammary cancer) ITGB1^−/−^ vs WT	C57Bl/6, female	LVH, LVSD	↑ tumour growth in WT, but not in ITGB1^−/−^	–	= proliferation of ITGB1^−/−^ and Renca cells upon incubation with serum from TAC‐operated mice	
Permanent LAD ligation (vs. sham surgery) TAC (vs. sham surgery)	CRC tumour model (germ‐free mice, AZM/DDS)	C57BL/6J, male	LAD: LVSD, fibrosis TAC: LVH, fibrosis	Foecal transplanation: ↑ tumour growth in AZM/DDS model	Dysbiosis, e.g.: ↓ Blautia ↑ Bacteroides	↑ proliferation in AZM/DDS colonic tumors	^116^
Permanent LAD ligation (vs. sham surgery)	4 T1 cells into the mammary fat pad (mammary cancer)	BALB/c, female	LVSD, fibrosis, congestion	↑ tumour growth and proliferation = lung and liver metastases	Cardiac remodelling → NGF	↑ proliferation of 4 T1 cells upon incubation with NGF, abrogated by silencing or GW441756	^117^
	4 T1 cells into the mammary fat pad (mammary cancer) + GW441756	BALB/c, female	LVSD, fibrosis, congestion	Reduced tumour growth and proliferation			
Permanent LAD ligation (vs. sham surgery)	E0771 cells into the mammary fat pad (mammary cancer)	C57BL/6J, female	No cardiac remodelling	↑ tumour growth and proliferation	↑ Ly6C^hi^ monocytes, which halt anti‐tumor immune responses	–	^118^
	MMTV‐PyMT (mammary cancer)			↑ tumour growth and proliferation ↑ dissemination to the lung			
Permanent LAD ligation (vs. sham surgery)	ApcMin/J mouse (intestinal adenoma)	C57BL/6J, male	LVSD, fibrosis, ↓ SBP, congestion	↑ tumour growth and proliferation	Cardiac remodelling → tumorigenic factors (SerpinA3)	↑ proliferation of HT29 colorectal cancer cells upon incubation with SerpinA3	^113^
Transplantation of infarcted heart (vs. transplantation of sham‐operated heart)			LVSD, fibrosis, no congestion	↑ tumour growth and proliferation			
Permanent LAD ligation (vs. sham surgery)	Renca cells into the kidney (renal cancer)	BALB/c, male	LVSD, fibrosis, no congestion	= tumour growth	–	= proliferation of Renca cells upon incubation with of SerpinA3 and other remodelling factors	^119^
αMHC‐tTA × ATF3	MMTV‐PyMT cells into the mammary fat (mammary cancer)	C57Bl6, female	LVH, LVSD, fibrosis	↑ tumour growth, and proliferation ↑ tumour fibrosis	Cardiac remodelling → tumorigenic factors (POSTN, CTGF, FN, SerpinE1, SerpinA3)	↑ proliferation of MMTV‐PyMT and LLC cells upon incubation with serum from ATF3‐transgenics	^120^
	LCC cells into the s.c. tissue of the flank (lung cancer)	C57Bl6, male	LVH, LVSD	↑ tumour growth			
	i.v. MMTV‐PyMT cells (cancer metastases)	C57Bl6, female	–	↑ lung metastases			
PE (10 mg/kg/day) for 4 weeks	MMTV‐PyMT cells into the mammary fat (mammary cancer)	C57Bl/6, female	LVH and fibrosis, no dysfunction	↑ tumour growth and proliferation	Cardiac remodelling → tumorigenic factors (POSTN, CTGF, FN)	↑ proliferation of MMTV‐PyMT cells upon incubation with serum from PE‐infused mice	^121^

αMHC‐Tta, ^α^ myosin heavy chain‐tetracycline transactivator; ApcMin/J, adenomatous polyposis coli mutant; ATF3, activating transcription factor 3; AZM/DDS, azithromycin/dexamethasone; CTGF, connective tissue growth factor; CRC, colorectal cancer; FN, fibronectin; ITGB1^−/−^, integrin beta knockout; i.v., intravenous; JDP2, Jun dimerization protein 2; LAD, left anterior descending coronary artery; LCC, large cell carcinoma; LLC, Lewis lung carcinoma; LVH, left ventricular hypertrophy; LVSD, left ventricular systolic dysfunction; Ly6C^hi^, lymphocyte antigen 6 complex, locus c high; MMTV‐PyMT, mouse mammary tumour virus‐polyoma middle T antigen; NGF, nerve growth factor; NOD/SCID, non‐obese diabetic/severe combined immunodeficiency; NS, not specified; PE, phenylephrine; POSTN, periostin; Postn^−/−^, periostin knockout; SBP, systolic blood pressure; s.c., subcutaneous; SerpinA3, serine protease inhibitor A3; SerpinE1, serine protease inhibitor E1; TAC, transverse aortic constriction; WT, wild‐type.

Preclinical models have further provided evidence for the role of secreted factors^113^ and the immune system^118^ as mediators between HF and cancer. Remodelling factors that have been suggested as mediators of the tumour‐supporting effect of HF include serpinA3, periostin, nerve growth factor, connective tissue growth factor, fibronectin, and inflammatory cytokines.^112^, ^113^, ^114^, ^115^, ^117^, ^120^, ^121^ Myocardial infarction (MI)‐induced epigenetic reprogramming of Ly6C^hi^ monocytes in the bone marrow reservoir to an immunosuppressive phenotype and their recruitment to tumours has also been shown to underlie MI‐induced tumour growth.^118^ However, HF induced by transverse aortic constriction surgery also promoted tumorigenesis in immunodeficient mice, indicating that immune dysregulation may not be essential.^114^ In addition, microbial dysbiosis, which often is observed in HF, is present in a mouse model of MI‐induced HF and transplantation of the ‘HF‐related microbiome’ into recipient mice accelerated colon tumour growth.^116^

In summary, preclinical models suggest that HF may promote cancer growth through secreted factors and inflammatory mediators, indicating the potential of available preclinical models to evaluate the anticancer effect of HF medication.^122^

## Novel insights from drugs with both cardioprotective and anti‐tumour activity

In addition to the intriguing concept that HF may be tumorigenic, the fact that patients with CVD also have a higher risk of cancer than that in the general population suggests potential common mechanisms and therapeutic targets.^123^, ^124^, ^125^ This notion has been supported by some recently developed anti‐tumour therapies, particularly those that target the NLRP3 inflammasome, such as NLRP3 and IL‐1 inhibitors, which have also been shown to protect the cardiovascular system.

Consistent with preclinical results obtained on the role of IL‐1 in tumour progression, IL‐1 receptor antagonists and anti‐IL‐1 monoclonal antibodies can inhibit primary tumour growth and metastasis in animal studies.^126^ Furthermore, genetic associations in cancer patients indicate that combining anti‐IL‐1 strategies with checkpoint blockade immunotherapy may benefit patients with haematological, pancreatic or breast cancer, and clinical trials are ongoing.^126^ As IL‐1 can stimulate CRS‐related toxicity of CAR‐T therapy, this combination might not only be effective against cancer but may also help to reduce the cardiovascular toxicity associated with cell therapies.^57^ The Canakinumab Anti‐Inflammatory Thrombosis Outcome Study (CANTOS), a large, randomized, double‐blind, placebo‐controlled phase 3 trial of canakinumab in patients with a history of MI with atherosclerosis,^127^ showed that canakinumab was associated with a dramatic (>60%) reduction in lung cancer incidence and mortality,^128^ specifically in molecular subtypes of lung cancer.^129^ Based on these results, single‐agent canakinumab has been prospectively investigated in a randomized, double‐blind, placebo‐controlled phase 3 trial in patients with surgically resected stage II–III non‐small cell lung cancer (NCT03447769). However, this trial (CANOPY‐A) did not show a benefit in disease‐free survival,^130^ and further results from other trials will follow.

NLRP3 inhibition is another therapy with potential combined anti‐tumour and cardioprotective properties. The NLRP3 inflammasome is a source of IL‐1β and IL‐18, and regulates pyroptosis, autophagy, and oxidative stress,^131^ as evidenced by the known anti‐inflammatory and cardiovascular protective properties of colchicine,^132^, ^133^ the oldest non‐specific inflammasome inhibitor. Colchicine is an effective treatment for pericarditis and may also reduce the risk of ischaemic cardiovascular events in patients with a recent MI, although at the expense of increased non‐CVD mortality.^134^ Beside preclinical evidence of its potential cardioprotective properties, and the clinical evidence of IL‐1 and IL‐6 inhibition against cardiovascular risk,^135^ direct clinical evidence of NLRP3 inhibition in protecting against HF or CVD is still lacking. Various clinical trials studying colchicine or IL‐1 inhibition in stable or acutely decompensated HF with reduced ejection fraction (reviewed in Olsen *et al*.^136^) have also failed to convincingly demonstrate a strong protective potential. On the other hand, targeting the NLRP3 inflammasome to further improve immunotherapy may be a promising therapeutic approach for prostate, breast, colorectal and lung cancer, as well as melanoma.^137^ However, NLRP3 may act as a double‐edged sword in carcinogenesis, exerting both positive and negative effects.^138^ More preclinical and clinical research is therefore needed to identify the best subgroup of cancer patients who will benefit from NLRP3 inhibition with common cardiovascular and cancer risk. This also applies to the danger associated with molecular pattern and activator of the NLRP3 inflammasome, S100A9,^139^, ^140^ which has an ambivalent role in cancer^141^ and CVD.^142^

In conclusion, specific inflammatory molecular pathways, particularly those related to the NLRP3 inflammasome, may serve as common therapeutic targets for both cancer and HF. However, further mechanistic validation in preclinical models is essential, especially given the unclear role of the NLRP3 inflammasome (and its drivers) in both tumorigenesis and CVD.

## Limitations of current models

### Gaps between preclinical modelling and clinical observations in human patients

The interpretation of data from preclinical modelling is crucial for avoiding translational failures.^143^ The aim is not only to avoid loss of time and the misallocation of funds and resources, but more importantly to avoid patients entering clinical phases that are doomed to fail or, conversely, to reduce the risk of promising new therapies being overlooked. Information obtained in the preclinical phase of a new chemical entity, or a new method, provides valuable insights during the clinical development phases. Ignoring or misinterpreting such signs has proven to be costly and even dangerous when candidate therapies enter clinical evaluation, leading to time and resources lost, and patients enrolled in fruitless trials. This underscores the importance of effective translational preclinical models and the need for precise preclinical model requirements to guide translational scientists.

Despite efforts to replicate the key features of human CTRCD, current preclinical models have significant limitations that need to be addressed in future research (*Table* *3*, *Figure* *1*). To accurately model CTRCD, it is essential to recognize that many anticancer treatments cause late‐onset cardiotoxicity, requiring the development of *in vivo* and *in vitro* models that reflect the progressive dysfunction observed in patients.^76^, ^144^, ^145^ This can be accomplished by mimicking clinical regimens with repeated exposures to low to medium doses of anticancer drugs or radiotherapy. Yet, a significant number of preclinical studies still utilize *in vivo* or *in vitro* systems exposed to a single high dose of anticancer treatment, which raises concerns about the translational relevance of the study conclusions. Another important aspect to consider in preclinical modelling of CTRCD is species selection. For instance, the use of inappropriate animal species, such as rodents, has led to confusion in studies investigating the cardiotoxicity of the humanized monoclonal antibody trastuzumab, which does not bind to the equivalent of the HER2 receptor (Neu) in rats or mice.^92^ Therefore, future research on the cardiac side effects of the emerging class of drugs known as antibody‐drug conjugates^156^ should involve careful species selection or the use of humanized models,^157^ particularly for those based on trastuzumab.

**Table 3:** Gaps in preclinical modelling and recommendations for future research

Topic	Gap in modelling	Recommendation for future research	Examples/reference
Late‐onset manifestation of CTRCD (especially anthracyclines and radiation‐induced cardiotoxicity)	*In vivo*: study of CTRCD in animals treated with a single, high dose of anticancer drug/radiation, usually leading to early and high mortality rates due to non‐cardiac toxicity.	Implement treatment protocols that closely replicate human therapeutic regimens in terms of administration route, number of cycles, dosage and pharmacokinetic profile.	Establishment of a murine model that provokes modest and progressive doxorubicin cardiotoxicity without constitutional symptoms, reminiscent of the effects seen in patients^144^
	*In vitro*: study of CTRCD in cells/slices/EHT following one dose of anticancer drug/radiation.	Implement repeated treatment schemes.	4‐hit model^76^ 2‐hit model^145^
Crosstalk between tumour cells and the myocardium in CTRCD	Study of CTRCD in non‐tumour‐bearing animals.	Perform treatment with anticancer drug/radiation in animals with tumour xenografts (syngeneic models or PDX in SCID mice) or in genetically engineered mice with spontaneous tumour growth.	AML cells killed by doxorubicin release IL‐1α leading to cardiac metabolic rewiring^146^ ICI‐induced LV dysfunction detected in tumour‐bearing mice only.^94^
	Study of CTRCD in non‐tumour comprising *in vitro* model systems.	Development and use of heart‐tumour chips.	A heart‐breast cancer‐on‐a‐chip platform for disease modelling and monitoring of cardiotoxicity induced by cancer chemotherapy.^147^
Interaction of the immune system with the myocardium (and/or the tumour) in CTRCD	*In vivo*: study of CTRCD in immunodeficient animals.	*In vivo*: use of immunocompetent animals.	Targeting early stages of cardiotoxicity from anti‐PD‐1 ICI therapy.^94^
	*In vitro*: study of CTRCD in cells/slices/EHT in the absence of immune cells.	*In vitro*: implement 2D and 3D co‐culture systems, including immune cells in addition to cardiac cells. Use of cardiac slices.	Effects of co‐cultured macrophages on iPSC proliferation, cardiac differentiation, and maturation.^77^
Intra‐ and inter‐species phenotypic variability of anticancer therapy cardiotoxicity	*Intraspecies variability*: the severity of cardiotoxicity phenotypes may vary based on the genetic background of the animal strain used, with potential differences even between the N and J substrains of C57BL/6 mice due to genetic variations.	Select sensitive strain backgrounds and/or optimizing treatment doses/regimens based on the sensitivity of the selected strain.	Strain background differences in the severity of cardiovascular phenotypes.^148^, ^149^
	*Interspecies variability*: rodents have been used to study trastuzumab cardiotoxicity although the drug does not bind rat or mouse *ErbB2/Neu*, generating results hard to interpret (off‐target rather than on‐target cardiotoxicity).	Select relevant binding species (e.g. human pluripotent stem cells or humanized models like transgenic animals expressing human HER‐2) to study HER‐2 inhibitors cardiotoxicity.	Trastuzumab, and by extension trastuzumab drug conjugates, do not bind rat or mouse *Neu*.^92^
Sex‐specific cardiovascular risks of cancer and its therapies	Sex‐based differences of cancer and cancer therapy cardiotoxicity are underexplored.	*In vivo*: evaluate anticancer therapies in both male and female animals of the same background, or in the sex specific to the cancer. Report the sex of the animals (ARRIVE guidelines).^150^	Sex‐specific cardiovascular risks of cancer and its therapies.^151^, ^152^, ^153^
		*In vitro*: evaluate anticancer therapies in both male and female *in vitro* model systems, or in the sex specific to the cancer. Report the sex of the cells/*in vitro* model used. Use of isogenic male vs. female iPSC‐derived CMs.	
Genetic predisposition to CTRCD	Need for model systems reflecting genetic predisposition to CTRCD.	Use of genomic editing of human iPSC for validation of significant variants identified in CGAS and GWAS studies.	Human *in vitro* models for assessing the genomic basis of CTRCD.^154^
Racial/ethnic disparity in CTRCD	Racial/ethnic disparity in the use of cells/*in vitro* systems for CTRCD.	Use of *in vitro* model systems with cells from patients with different ethnic background.	Racial disparity in anthracycline‐induced CTRCD.^155^

2D, two‐dimensional; 3D, three‐dimensional; AML, acute myeloid leukaemia; ARRIVE, animal research, reporting of *in vivo* experiment; CGAS, candidate gene association study; CM, cardiomyocyte; CTRCD, cancer therapy‐related cardiac dysfunction; EHT, engineered heart tissue; GWAS, genome‐wide association study; HER‐2, human epidermal growth factor receptor 2; ICI, immune checkpoint inhibitor; IL‐1α, interleukin‐1α; iPSC, induced pluripotent stem cell; LV, left ventricular; PD‐1, programmed cell death protein 1; PDX, patient‐derived xenograft; SCID, severe combined immunodeficiency.

Efforts should also focus on modelling inter‐organ interactions, which are increasingly recognized as crucial in the pathogenesis of CTRCD. In this respect, despite increasing political pressure to reduce animal research in the European Union, animal models remain indispensable in basic cardio‐oncology studies. They play a crucial role in the translational pathway, providing the only means of gaining essential pathophysiological insights into disease mechanisms at both (inter)‐organ and systemic level.^98^ Specifically, interactions between the myocardium and the primary target of anticancer therapies, the tumour, are essential to understand. While most basic cardio‐oncology research is conducted using tumour‐free models, growing evidence suggests that CTRCD can arise not only from the direct toxic effects of anticancer drugs on cardiac cells, but also from effects mediated through the tumour. Consequently, the effects of cancer therapy can vary depending on whether it is administered to tumour‐free or tumour‐bearing animals. For example, ICI therapy has been shown to induce left ventricular dysfunction in mice transplanted with syngeneic PD1‐sensitive melanomas but not in tumour‐free animals.^94^ Therefore, employing tumour‐bearing models is advised to more accurately replicate all factors involved in CTRCD pathogenesis (*Table* *3*). In large animals, this approach is rarely feasible due to the lack of syngeneic tumour cell lines and immunocompromised strains. This limitation requires the use of immunosuppressive therapy, which can lead to severe and difficult‐to‐manage complications,^158^ while also altering the effects of drugs or interventions. This limitation is effectively addressed by murine xenograft models, which utilize either syngeneic tumour cell implantation or human tumour cell inoculation in immunocompromised mice, as well as genetically engineered mice with spontaneous tumour growth (*Table* *2*). Additionally, *in vitro* heart‐tumour chips offer a promising alternative for studying these tumour–heart–drug interactions.

The immune system also plays a critical role in interacting with the myocardium and contributing to the development of CTRCD. However, immune cells are still rarely incorporated into *in vitro* models of CTRCD, highlighting the need for a more comprehensive use of cardiac slices and 2D and 3D co‐culture systems including inflammatory cells.^77^

Finally, as previously pointed out, recognizing early warning signs in the translational phase is essential for drug development in cardiovascular medicine. Ignoring these signs could become costly and dangerous when candidate therapies move into clinical evaluation.^143^

In conclusion, growing evidence suggests that genetic,^159^ gender,^151^ and ethnic^155^ factors influence predisposition to CTRCD. While groundbreaking research using genomic editing of human iPSC have started validating significant variants identified in candidate‐gene and genome‐wide association studies,^160^, ^161^, ^162^ gender and ethnic considerations have been largely overlooked in preclinical modelling of CTRCD. Therefore, future research should address these neglected aspects by increasing the inclusion of both male and female animals as well as utilizing *in vitro* model systems with cells from patients of diverse ethnic backgrounds.

### Impact of limitations on regulatory paths and full development process

Pathways for drug approval are becoming increasingly complex, with both the European Medicines Agency and US Food and Drug Administration (FDA) focusing on accelerated approval pathways. Regulatory agencies face the challenge of balancing the demand for rapid access to new and effective drugs with the need for comprehensive safety and efficacy data prior to patient use. Unfortunately, drug development regulations in different therapeutic areas are far from harmonized, and this affects the number of new chemical entities reaching the market.^163^ As an example, under the FDA's new ‘breakthrough therapy’, designation of the approvals granted from 1987 to 2017, approximately 50% were for oncology, 20% for infectious diseases, and none for CVDs.^164^ There is no doubt that many CVDs, including the acute or chronic consequences of cancer treatment, should be considered severely‐debilitating or life‐threatening diseases, and it stands to reason that the approval of severely‐debilitating or life‐threatening disease therapeutics should parallel that of advanced oncology drugs. However, although the FDA provides regulatory guidelines for advanced cancer therapies,^165^ there is minimal FDA guidance for the early development of drugs for severely‐debilitating or life‐threatening diseases for which there are few available therapeutic options. Consequently, from an industry business perspective, drug development projects in CVDs could become less attractive, which may contribute to the diversion of funds to other areas deemed more lucrative. A negative trend can be already seen in the percentages of new CVD treatment over the total approval by FDA in the latest decades: between 2000 and 2017, in fact, the approved CVD therapies over the total of the new approvals by FDA declined from 9.09% to 5.29%.^166^ Industry‐funded development research will just focus elsewhere if the trend continues.

From an academic perspective, several factors can hinder or slow down the development process, including limited awareness and infrastructure for patenting, spin‐offs, and industry partnerships. Researchers often prioritize publishing over commercial applications, missing opportunities to translate innovations into practical solutions, particularly in fields like life sciences where intellectual property protection is crucial. Many lack knowledge of the patenting process, including optimal timing and procedures. While universities have technology transfer offices, their effectiveness varies, and structured pathways for spin‐offs are often lacking. Early industry collaborations, which provide funding and expertise, are underutilized due to limited connections and concerns over research independence. Strengthening academia–industry networks, improving intellectual property training, and fostering entrepreneurial support would help streamline the development process.^167^, ^168^

For these reasons, we cannot emphasize enough the need for continuous and open discussions between translational researchers, trialists, pharmaceutical industry representatives and regulatory authorities to ensure that cardio‐oncology is properly positioned in the regulatory compass and, overall, in the world health‐economic compass.

## Conclusions and future directions

Over the past decade, cancer therapy has experienced a paradigm shift from non‐specific cytotoxic chemotherapies, such as anthracyclines, to more targeted treatments including monoclonal antibodies, TKIs, ICIs, and cell therapies. While these advancements have led to better clinical outcomes for patients with both solid and haematological malignancies, they also come with a broad spectrum of side effects, including cardiac toxicity. Consequently, the development of new anticancer drugs presents the challenge of uncovering the mechanisms underlying the relative CTRCD, which necessitates the creation of effective preclinical disease models. Although a combination of preclinical *in vitro* and *in vivo* models across various species has provided a comprehensive understanding of the cardiotoxicity associated with conventional anticancer therapies, the cardiac effects of some newer treatments are only beginning to be explored, while others remain largely unexplored and warrant future research. For instance, further investigation is required into the cardiac impact of cyclin‐dependent kinase 4/6 inhibitors^169^ or the antibody‐drug conjugates – an innovative and evolving class of antineoplastic agents that combine monoclonal antibodies with biologically active drugs.^156^ Furthermore, the potential interactions among cancer therapies in multimodal regimens have been insufficiently addressed in preclinical modelling, and thus warrant the attention of future studies.

The development of new immunotherapies, coupled with the growing recognition of the pivotal role of the immune system in CTRCD, and as a common denominator in cancer and HF,^170^ underscores the importance of using immunocompetent animals and multicellular 2D and 3D model systems, encompassing, in addition to cardiomyocytes, fibroblasts (which possess inflammatory properties)^171^ and immune cells. The mutual interaction between the tumour and the heart further supports the use of tumour‐bearing animals^94^ and of heart‐tumour‐on‐a‐chip platforms.^147^ Genetic predisposition to^154^ and sex‐based differences in CTRCD^151^ also highlight the importance of genomic editing of human iPSC for validation of significant genetic variants associated with CTRCD and the testing of anticancer therapies in both male and female cells/model systems.^152^ Racial/ethnic disparity in CTRCD emphasizes the need for *in vitro* model systems with cells from patients with different ethnical background.^155^ Finally, in view of personalized medicine, patient‐derived model systems may serve as predictive models of CTRCD.^172^ Beyond the recognized use of patient‐specific iPSC‐CM and multicellular model systems, the use of cardiac slices derived from endomyocardial biopsies holds promise.

In conclusion, the emergence of novel anticancer drugs, the recognition of the role of the immune system in CTRCD, our increased understanding of the reciprocal relationship between the heart and cancer, and the growing appreciation for sex‐based and ethnic disparities of CTRCD are driving the development of novel, improved preclinical model systems and approaches to close the gap between preclinical models and clinical observations in patients.
